# Direct-to-catheter ablation versus second line catheter ablation for persistent atrial fibrillation: Effect on arrhythmia recurrence, AF burden, early left atrium remodeling and quality of life

**DOI:** 10.1007/s10840-024-01916-6

**Published:** 2024-09-12

**Authors:** Hadi Younes, Besim Ademi, Eli Tsakiris, Han Feng, Amitabh C. Pandey, Mario Mekhael, Charbel Noujaim, Chanho Lim, Lilas Dagher, Abdel Hadi El Hajjar, Ghassan Bidaoui, Mayana Bsoul, Ala Assaf, Swati Rao, Christian Mahnkopf, Ghaith Shamaileh, Omar Kreidieh, Abboud Hassan, Yinshuo Liu, Yishi Jia, Francisco T. Polo, Nassir F. Marrouche, Eoin Donnellan

**Affiliations:** 1 Tulane Research Innovation for Arrhythmia Discovery (TRIAD), Cardiac Electrophysiology, Tulane University School of Medicine, New Orleans, LA USA; 2 Department of Cardiology, Klinikum Coburg, Coburg, Germany; 3 Medical School, University of Split, 21000 Split, Croatia

**Keywords:** Direct-to-catheter ablation, Second-line Ablation, History of anti-arrhythmic drugs, Persistent atrial fibrillation

## Abstract

**Background:**

Catheter ablation has obtained class 1 indication in ablation of young, healthy patients with symptomatic paroxysmal atrial fibrillation (AF). Anti-arrhythmic drugs (AADs) remain first-line therapy before ablating persistent AF (PersAF). We sought to evaluate the efficacy of a direct-to-catheter ablation approach against catheter ablation post AADs in PersAF.

**Methods:**

In this DECAAF II subanalysis, patients were stratified into two subgroups: ‘Direct-to-catheter’ group comprising patients who had not received AADs prior to ablation, and’second-line ablation’ group, comprising patients who had been on any AAD therapy at any time before ablation. Patients were followed over 18 months. The primary outcome was AF recurrence. Secondary outcomes included AF burden, quality of life (QoL) that assessed by the AFSS and SF-36 scores, and changes in the left atrial volume index (LAVI) assessed by LGE-MRI scans.

**Results:**

The analysis included 815 patients, with 279 classified as’direct-to-catheter’ group and 536 classified as’Second-line ablation’ group. The primary outcome was similar between both groups (44.8% vs 44.4%, p > 0.05), as was AF burden (20% vs 16%, p > 0.05). Early remodeling, reflected by LAVI reduction, was similar between the groups (9.1 [1.6—18.0] in the second-line ablation group and 9.5 [2.5—19.7] in the direct-to-catheter group, p > 0.05). QoL pre/post ablation was also similar (p > 0.05). On multivariate analysis, history of AAD was not predictive of AF recurrence(p > 0.05).

**Conclusion:**

Prior AAD therapy demonstrated minimal impact on atrial remodeling and QoL improvement, in addition to limited benefit on AF recurrence and burden post-ablation in patients with PersAF. Additional studies are warranted to explore the efficacy of catheter ablation as a first-line therapy in PersAF.

**Graphical Abstract:**

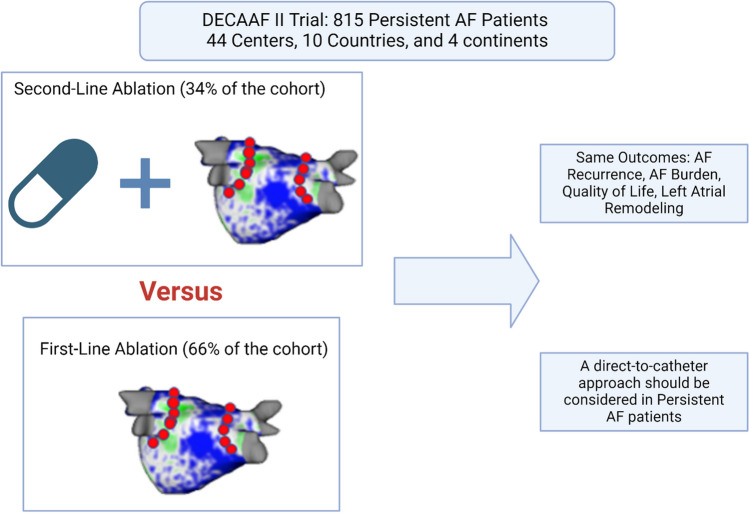

## Introduction

Atrial fibrillation (AF) is the most common sustained arrhythmia, affecting over 35 million patients globally and contributing significantly to an increased strain on the healthcare system[1]. Its disease course is progressive and can be complicated by acute, debilitating exacerbations, cardioembolic stroke as well as development of heart failure. These are directly related to an increase in morbidity and mortality [2].

Medical management of AF can be divided into two main categories, rhythm or rate control. While rate control has been historically considered equivalent to rhythm control, growing evidence demonstrates the importance of maintenance of normal sinus rhythm. Antiarrhythmic drugs (AADs) and catheter ablation are the two mainstay therapies in addressing AF rhythm.

The 2023 AHA/ACC/HRS guidelines regarding persistent atrial fibrillation (PeAF) management recommend AADs as first line therapy. Rhythm control agents such as amiodarone, flecainide and sotalol remain commonly used for medical management of AF. However, these medications have significant adverse reactions and side effect profiles that warrant careful clinical consideration and monitoring. These include but are not limited to pulmonary and thyroid toxicity, prolongation of the QTc interval as well as fatal proarrhythmic events[3]. The efficacy of these therapies also widely varies and is dependent on a multitude of factors such as age, co-morbidities, medication interactions and underlying left atrial size.

Catheter ablation has been shown to be an effective and safe treatment modality in patients with AF, while complications, such as cardiac tamponade, stroke and mortality, continue to decrease with time [4, 5]. Moreover, growing evidence exists for the preferential direct-to-catheter approach in the treatment of AF, circumventing an initial antiarrhythmic trial. As supported by a class 1 indication in the 2023 ACC/AHA guidelines, young and healthy patients with paroxysmal atrial fibrillation (PAF) may have lower recurrence rates with a direct-to-catheter ablation as compared to AAD therapy[3]. However, studies assessing direct-to-catheter approach in more advanced disease, PeAF, are lacking. To further investigate the benefit of a direct-to-catheter approach in the treatment of PeAF we sought to compare patients who were AAD-Naïve and underwent a direct to catheter approach compared to patients who underwent catheter ablation with a history of AAD use.

## Methods

### DECAAF II design

DECAAF II is a randomized multi-center clinical trial that included 843 patients with PeAF who were randomly assigned to PVI plus MRI-guided atrial fibrosis ablation (421 patients) or PVI alone (422 patients). Patients were followed over a period of 12–18 months using a mobile single lead ECG monitoring device to observe AF recurrence and burden. Patients were instructed to send one ECG recording from the device each day during the study period through a smartphone application. LGE-MRIs were preformed prior to ablation and at 3-months after ablation. Quality of life was assessed at baseline and 12 months using standardized Short Form Health Survey (SF-36) and the Atrial Fibrillation Survey Scale (AFSS).

### Sub-analysis design

In the DECAAF II subanalysis, patients were categorized based on their history of AAD use. This classification aimed to evaluate the influence of AAD therapy on treatment outcomes, especially investigating whether pre-ablation AAD administration yields additional benefits in AF management.

### Ablation procedure

In the study, patients were treated with either fibrosis-guided ablation approach plus Pulmonary Vein Isolation (PVI) or PVI alone. The primary tools used in these procedures were radiofrequency ablation (RFA) and cryoablation catheters. For those in the fibrosis-guided group, the process involved merging processed delayed-enhancement MRI images with a 3D mapping system during the ablation. This technique was essential for targeting fibrotic areas identified on the MRI. In contrast, the PVI group focused on the electrical isolation of all pulmonary veins, as per the Heart Rhythm Society Consensus Statement. If normal sinus rhythm was not restored post-PVI, even after cardioversion, additional measures were available to address recurrent arrhythmias.

### Antiarrhythmic drugs

In this study, physicians were advised against prescribing AADs following the ablation procedure. Patients with any history of AAD treatment prior to ablation were categorized as the 'Second-line ablation’ group. Those who did not have any history of AAD were categorized as’Direct-to-catheter’ group. The main AADs considered in this study included Amiodarone, Dofetilide, Dronedarone, Flecainide, Propafenone, and Sotalol. An analysis regarding the individual effect of each AAD on AF recurrence was also conducted.

### Primary and secondary outcomes

The primary end point of the study was the first confirmed recurrence of atrial arrhythmia (including AF, atrial flutter, or atrial tachycardia) lasting for at least 30 s after the 90-day blanking period, demonstrated by at least 2 consecutive 1-lead smartphone ECG device tracings, 1 positive reading on a clinical 12-lead ECG tracing, ambulatory monitor, or if the patient underwent repeat ablation. The daily smartphone ECGs were intended as the primary method for assessing atrial arrhythmia recurrence, but clinical and ambulatory ECGs served as back-up methods for detecting recurrence in patients who failed to reliably transmit smartphone ECG readings. A core laboratory at the University of Washington adjudicated the ECG findings. This DECAAF II substudy utilized the Toronto Atrial Fibrillation Symptom Severity Scale (AFSS) and SF-36 score as the main subjective outcome metrics to evaluate quality of life at baseline as well as at 12 months.

### Imaging

Patients underwent a delayed-enhancement MRI within 30 days prior to the ablation procedure using the Merisight delayed- enhancement MRI protocol (MARREK Inc) and at 3 months post-ablation. In this study, baseline MRI results were utilized to quantify left atrial (LA) fibrosis and LA volume in all patients, both at baseline and at the 3-month follow-up. Left Atrial Volume was indexed to body surface area.

### Statistical analysis

Variables of interest were compared among the two study groups. We checked the normality assumptions of all continuous variables studied in this work through the Shapiro–Wilk tests. Based on whether the normality assumptions were violated or not, Kruskal–Wallis tests and t-tests were conducted accordingly to the variables. In the meantime, they are presented through median (IQR) and mean (SD) in the text accordingly. Categorical variables were tested through Chi-square tests and were presented using counts (%). Kaplan–Meier curve was conducted on the primary outcome after ablation to compare the effects of different treatment strategies (direct-to-ablation vs second-line ablation), the corresponding p-value was obtained by the log-rank test. Further, a multivariable Cox model was developed to assess the group effects adjusting for other clinically relevant factors.

## Results

### Baseline characteristics

There were 279 patients in the Direct-to-catheter group and 536 in the Second-line ablation group. The average age was similar between the groups, with Group 1 averaging 62.1 years and Group 2, 62.0 years (p = 0.92). Sex distribution was also comparable, with females making up 21.1% of the Direct-to-catheter group and 20.5% of the Second-line ablation group. There was no significant difference within the groups in terms of treatment arms (In the direct-to-catheter group, 130 patients [46%] underwent pulmonary vein isolation; in the second-line ablation group, 278 patients [52%] underwent pulmonary vein isolation, p = 0.15). There were no significant differences between the groups in terms of the presence of comorbidities such as congestive heart failure (CHF), hypertension (HTN), diabetes mellitus (DM), previous stroke, vascular disease VascularDx, tobacco use, coronary artery disease (CAD), coronary artery bypass grafting (CABG), mitral valve disease. Hyperlipidemia was more prevalent in the second-line ablation group (36.5% vs 28.8%, p = 0.03) (Table 1).

**Table 1:** Baseline Characteristics

Demographics	Direct-to-catheter (N = 279)	Second-line ablation (N = 536)	Total (N = 815)	p value
Age, years (Mean ± SD)	62.1 ± 9.4	62.0 ± 9.0	62.0 ± 9.1	0.92
Sex (Female)	59 (21.1%)	110 (20.5%)	169 (20.7%)	0.83
Comorbidities				
CHF (Yes)	45 (16.1%)	109 (20.3%)	154 (18.9%)	0.15
HTN (Yes)	168 (60.2%)	312 (58.2%)	480 (58.9%)	0.60
DM (Yes)	23 (8.2%)	59 (11.0%)	82 (10.1%)	0.21
Stroke (Yes)	22 (7.9%)	47 (8.8%)	69 (8.5%)	0.70
Vascular Dx (Yes)	35 (12.5%)	46 (8.6%)	81 (9.9%)	0.07
Tobacco Use (Yes)	100 (35.8%)	205 (38.2%)	305 (37.4%)	0.50
CAD (Yes)	40 (14.3%)	62 (11.6%)	102 (12.5%)	0.26
CABG (Yes)	3 (1.1%)	9 (1.7%)	12 (1.5%)	0.50
Mitral Valve Dx (Yes)	17 (6.1%)	30 (5.6%)	47 (5.8%)	0.77
Hyperlipidemia (Yes)	82 (29.4%)	197 (36.8%)	279 (34.2%)	0.04
CHA2DS2-VASc Score median (Q1, Q3)	2.0 (1.0, 3.0)	2.0 (1.0, 3.0)	2.0 (1.0, 3.0)	0.85

Congestive Heart Failure (CHF); Hypertension (HTN); Diabetes Mellitus (DM); Stroke; Vascular Disease (Vascular Dx); Coronary Artery Disease (CAD); Coronary Artery Bypass Graft (CABG); Mitral Valve Disease (Mitral Valve Dx)

### Compliance rates and primary outcome

**Fig. 1:**
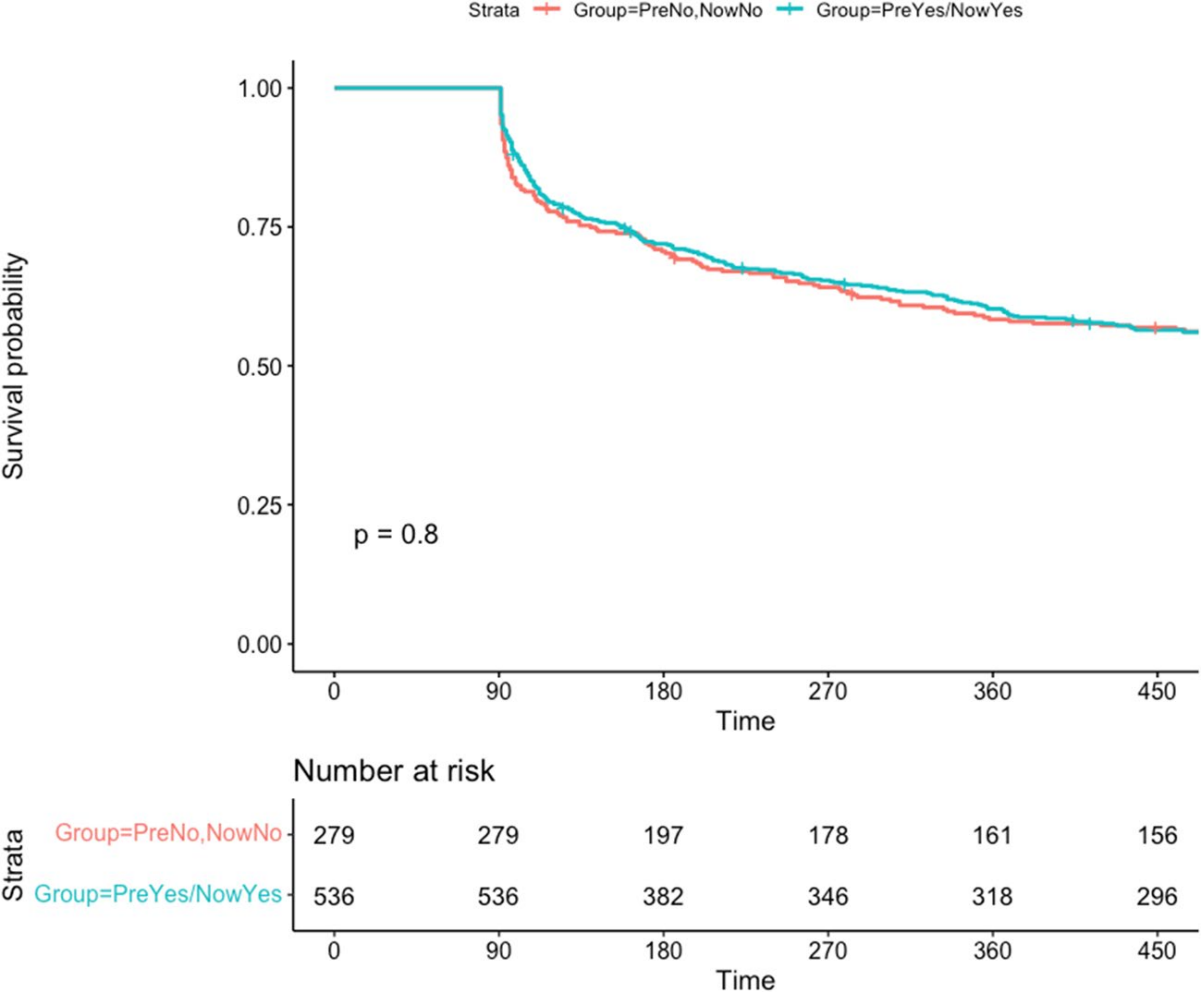
Time-to-primary outcome analysis

Within the 3-month blanking period, the compliance rates are 33 ± 30% and 32 ± 30% (p = 0.24) for the Direct-to-catheter group and Second-line ablation respectively. Within the post blanking period, the compliance rates are 30 ± 28% and 26 ± 26% (p = 0.054) for the Direct-to-catheter group and Second-line ablation groups respectively. The rates of the primary outcome were identical in both groups, with 44.8% in the direct-to-catheter group and 44.4% in the Second-line ablation group, showing no statistical difference (p = 0.91; Fig. 1). Similarly, there was no difference in the time to primary outcome between both groups (285 days for direct-to-catheter group versus 288 days for Second-line ablation group, p = 0.79). The post-ablation arrhythmia burden at 12 months was also similar between the two cohorts (20% for direct-to-catheter vs 16% for second-line ablation, p = 0.08).

### Multivariate analysis

In the assessment of factors contributing to AF recurrence, a Cox proportional hazards regression analysis was conducted. The analysis included the following covariates: medication status (History of AADs), patient age (AgeYrs), sex, CHF, HTN, DM, history of stroke, VascularDx, tobacco use, CAD and hyperlipidemia. Age, LA volume and sex were found to be a significant predictor of the outcome. In contrast, the history of AADs use was not a significant predictor of outcome (p > 0.05). The analysis of the other covariates in the model did not reach statistical significance, suggesting no evidence to indicate that these factors independently affected the outcome in this cohort (Table 2).

**Table 2:** Multivariate analysis of the primary outcome of interest

	Coefficient	SE	P-value	Hazard Ratio	95% CI
History Of AAD	-0.002	0.118	0.99	1.00	0.79; 1.26
Years_Diag_To_Ablation	0.02	0.013	0.18	1.02	0.99; 1.04
La_Volume	0.01	0.001	< 0.001	1.01	1.01; 1.02
Age	0.01	0.007	0.49	1.01	0.99; 1.02
Sex Male	-0.52	0.142	< 0.001	0.60	0.45; 0.79
DM	-0.27	0.204	0.19	0.76	0.51; 1.14
HTN	-0.17	0.119	0.18	0.84	0.67; 1.08
Stroke	0.23	0.196	0.25	1.25	0.85; 1.84
Vasculardx	-0.09	0.231	0.70	0.92	0.58; 1.44
Tobacco	0.14	0.118	0.24	1.15	0.91;1.45
CAD	-0.01	0.207	0.95	0.99	0.6584
Hyperlipidemia	0.14	0.124	0.26	1.15	0.902
CHF	0.05	0.146	0.74	1.05	0.7892

Diabetes Mellitus (DM); Hypertension (HTN); Vascular Disease (Vascular Dx); Coronary Artery Disease (CAD); Congestive Heart Failure (CHF)

### Secondary outcomes

#### Quality of life and early remodeling

**Fig. 2:**
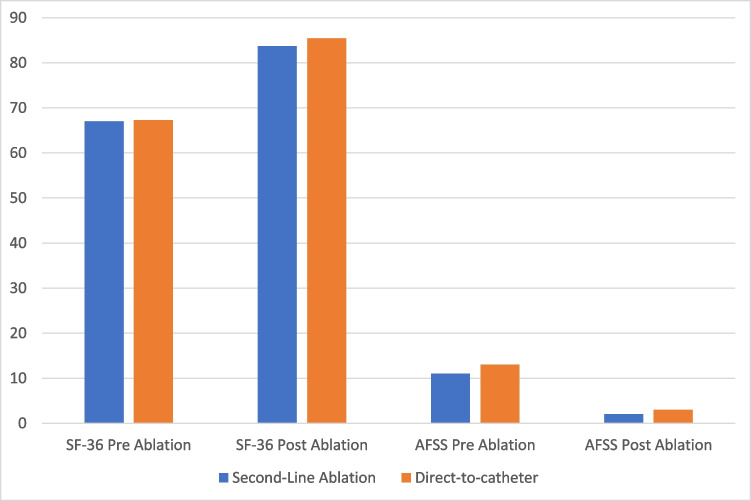
Quality of Life using the SF-36 and AFSS score compared between the 2 groups before and after ablation. No Statistical Difference Was Achieved

In this post-hoc analysis, the Quality-of-Life outcomes measured by SF-36 and AFSS scores were compared between the two groups before and after ablation. The SF-36 scores pre-ablation were 67.0 (49.3—79.4) for the second-line ablation group and 67.3 (53.0 – 81.0) for the direct-to-catheter group, with no significant difference (p = 0.28). Post-ablation, the scores increased to 83.7 (69.1—91.8) and 85.4 (72.3—92.0) respectively, still showing no significant difference (p = 0.18). AFSS scores pre-ablation were 11.0 (5.0—18.0) for the second-line ablation group and 13.0 (6.0—19.0) for the direct-to-catheter group (p = 0.09), and post-ablation, the two groups reported respectively the scores of 2.0 (0.0—8.0) and 3.0 (0.0—7.0), again with no significant difference (p = 0.21; Fig. 2). Additionally, the change in SF-36 and AFSS scores from baseline to post-ablation was statistically significant in both groups. More specifically, median SF-36 increase in the “second-line ablation” group is 12.1 (3.4—26.3; p < 0.001) and in the “direct-to-catheter” group is 12.7 (2.4—24.9; p < 0.001) but no difference among the two groups (p = 0.86). The median AFSS decrease in the “second-line ablation” group is 6.0 (1.0—12.0; p < 0.001) and 6.0 (1.0—12.0; p < 0.001) in the “direct-to-catheter” group with no significant differences among the two groups as well (p = 0.79).

The Left Atrial Volume Index (LAVI) reduction indicated early remodeling with reductions of 9.1 (1.6, 18.0)in the second-line ablation group and 9.5 (2.5, 19.7) in the direct-to-catheter group, without a significant difference (p = 0.11; Table 3).

**Table 3:** Early Remodeling reflected by the LA volume indexed to Body Surface Area as assessed by LGE-MRI and compared between the 2 groups

	Second-line ablation	Direct-to-catheter	P-value
Pre-Ablation LAVI	59.3 (47.8, 71.8)	62.2 (51.1, 75.9)	0.02
Post-Ablation LAVI (ml/m^2^)	47.9 (40.4, 57.3)	50.7 (40.3, 62.3)	0.10
LAVI reduction (ml/m^2^)	9.1 (1.6, 18.0)	9.5 (2.5, 19.7)	0.11

LAVI: Left Atrial Volume Indexed to Body Surface Area

#### Impact of individual antiarrhythmic drugs on AF recurrence rates

We examined the relationship of amiodarone (n = 144), dofetilide (n = 23), dronedarone (n = 14), flecainide (n = 90), sotalol (n = 70) with the primary outcome. On multivariate analysis, each individual AAD used was not predictive of AF recurrence. (Table 4).

**Table 4:** Multi-variate analysis of the primary outcome. No statistical difference in each of the individual anti-arrhythmic medications

	Coefficient	SE	P-value	Hazard Ratio	95% CI
Amiodarone	-0.03	0.14	0.821	0.97	0.75;1.28
Dofetilide	0.16	0.30	0.599	1.17	0.65; 2.09
Dronedarone	-0.36	0.45	0.428	0.70	0.29;1.70
Flecainide	-0.07	0.17	0.667	0.93	0.67;1.30
Sotalol	-0.15	0.20	0.444	0.86	0.58;1.30

## Discussion

To our knowledge, this analysis is novel and presents a direct comparison of AAD naïve ablation versus AAD positive ablation patients in PeAF. In our post-hoc examination of the DECAAF II study data, no discernible differences were observed in AF recurrence or burden post-ablation in regard of AAD use. Quality of life measures and indicators of early left atrial remodeling also appeared unaffected by AAD administration before or at the time of ablation. These findings suggest that catheter ablation may be equally effective as a primary intervention for PeAF, without the necessity of preceding AAD therapy that might not add a benefit in terms of outcomes.

The consideration of side effects, drug interactions and monitoring necessary to pursue AF therapy with AADs are not insignificant. The interplay of polypharmacy and the complex nature of drug interactions often results in adverse effects from AADs, despite efforts to mitigate such occurrences, Recently, a 2024 observational study of 770,000 patients in the Korean Health System found that AAD use in AF, while decreasing the risk of major cardiovascular events, actually increased the risk of syncope, bradyarrhythmia and pacemaker implantation[6]. Oppositely, adverse events from catheter ablation is extremely uncommon, and rates continue to decrease as the procedure matures and is optimized [5]. Our current analysis expands our current knowledge beyond paroxysmal AF, demonstrating that even patients with a more advanced arrhythmia burden may experience favorable cardiac remodeling and a higher likelihood of arrhythmia-free survival with catheter ablation of AF. This underscores the potential of ablation as a beneficial primary intervention for a more advanced disease AF population.

It is important to note that other studies have shown catheter ablation to improve quality of life as well as beneficial remodeling of the left atrium in AF patients [7]. The EARLY-AF and STOP AF trials have showcased the superiority of ablation, specifically using cryoablation, as a first-line therapy over medical management with a significant reduction in AF recurrence [8, 9]. Additionally, the extended follow-up study of EARLY-AF indicated that ablation may slow the progression from paroxysmal to persistent AF more effectively than medical therapy [10]. Our analysis examines outcomes of radiofrequency ablation but our results demonstrating the advantage of ablation are coherent with these previous trials. The CASTLE-AF trial demonstrated the superiority of catheter ablation over medical management of AF patients with heart failure. While our analysis did not exclusively examine HF patients, peAF is known to be a potent risk factor for development of HF. Winkle et al.’s study of 1504 ablations found that patients who failed increasingly more AADs had worse ablation outcomes, citing AF progression during the AAD trials as a possible explanation [11]. In patients with advanced AF, we believe that a prompt ablation may logically improve outcomes, as short term medical management before eventual ablation may only allow for further progression of the AF towards a sicker heart such as heart failure. A trend favoring early ablation is directly reflected in the most recent AHA/ACC guidelines giving a type 1 indication for ablation as first line therapy in young and healthy patients with paroxysmal AF [3]. Our analysis is coherent with this upgraded indication, and further calls into question the utility of medical management for peAF and supports first-line ablation for this population. While this DECAAF II subanalysis does have limitations as described below, the lack of benefit from AAD use is compelling to further investigate consideration of catheter ablation as first line therapy in PeAF. In conclusion, we believe that the common practice of AAD trial before ablation, at best, may not provide any benefit and at worst, could allow for further progression of AF before definitive procedural treatment.

## Limitations

A limitation of this study is its retrospective design and the underrepresentation of female and African American patients, which may limit the applicability of the results to the entire AF patient population. Additionally, the exact duration of AAD usage before ablation is unknown, along with details on AAD dosage, sinus rhythm before ablation, and treatment adherence. Further investigation is needed to determine the superiority of specific ablation techniques and protocols, as well as ensure its long term superiority over medical management.

## Conclusion

Catheter ablation may be a viable first-line therapy for PeAF, limiting the exposure to AAD medication side effects and adverse events. Further research should be conducted to evaluate the efficacy of catheter ablation as compared to AAD use in patients with PeAF.

## Data Availability

The data supporting the findings of this study are available from the DECAAF II trial and can be obtained from the corresponding author upon reasonable request.
